# A Rare Case of Unilateral Auditory Neuropathy Induced by Proton Therapy

**DOI:** 10.7759/cureus.45085

**Published:** 2023-09-12

**Authors:** Mathilde Hoornaert, Celine Laurent, Clement Lelong, Paul Deltenre, Benoit Devroede

**Affiliations:** 1 Otolaryngology-Head and Neck Surgery, Erasme Hospital, Brussels University Hospital (HUB), Brussels, BEL; 2 Auditory Neurophysiology Laboratory, Brugmann Hospital, Brussels, BEL; 3 Otolaryngology-Head and Neck Surgery, Queen Fabiola Children's University Hospital, Brussels University Hospital (HUB), Brussels, BEL

**Keywords:** case report, proton therapy, radiation therapy, hearing loss, auditory neuropathy

## Abstract

Hearing loss (HL) is one of the most common complications of the treatment in head and neck oncology. Most cases of HL are due to the ototoxicity of platinum-based chemotherapy (PBC) - resulting usually in a symmetric bilateral sensorineural hearing loss (SNHL) - or radiotherapy. Radiation-induced SNHL is progressive, permanent, and dose-dependent. Total dose and follow-up time are important factors affecting incidence rates. However, the hearing consequences of proton radiation therapy (PRT), a radiation-type therapy especially used in pediatric malignancies of the central nervous system (CNS), remains unclear and poorly documented. We report here a case of a four-year-old patient with unilateral auditory neuropathy spectrum disorder (ANSD) related to PRT. This case highlights the need for appropriate auditory monitoring in patients undergoing PRT for CNS or head and neck malignancies.

## Introduction

Hearing loss (HL) is a common complication of cancer treatments, particularly in head and neck malignancies. They generally stem from a direct impact on the sensory cells of the Corti's organ, caused by the use of platinum-based chemotherapy (PBC) and resulting in an irreversible bilateral high-frequency sensorineural hearing loss (SNHL) [1,2]. Conversely, auditory neuropathy spectrum disorder (ANSD, a diverse set of impairments that may affect different auditory structures from inner hair cells to auditory nuclei) is rarely associated with anticancer treatments. ANSD is defined from an electrophysiological perspective by the persistence of responses from the external cochlear hair cells in the absence of neural responses during the recording of brainstem auditory evoked potentials (BAEP)[3]. This cochlear response can be highlighted by the presence of acoustic otoemissions and/or the presence of a cochlear microphonic potential in BAEPs.

We present a case of unilateral ANSD associated with PRT in a young patient with an atypical teratoid/rhabdoid tumor (AT/RT) of the posterior fossa. AT/RT is a rare aggressive central nervous system (CNS) tumor that typically affects children under the age of three and is associated with an unfavorable prognosis [4]. The gold standard treatment for these tumors includes gross total resection and postoperative photon radiotherapy (XRT) or PRT combined with chemotherapy [5]. Technological advances in radiotherapy planning and delivery are reducing the exposure of normal tissues, leading to improved toxicity outcomes. Besides continuous advances in image-guided (IG), intensity-modulated (IM) XRT, particle therapy with protons is establishing itself as a high-conformal radiotherapy modality that can improve normal tissue dose sparing while maintaining excellent target coverage. Indeed, thanks to the physical characteristics of protons, such as the typical dose distribution within the *Bragg peak*, PRT could represent a safe alternative to XRT for pediatric tumors or other neoplasms arising next to critical organs at risk (OARs). Nevertheless, radiobiological uncertainties about the interaction of these charged particles with normal and neoplastic cells persist [6]. However, its effect on auditory functions remains largely unknown.

## Case presentation

**Figure 1:**
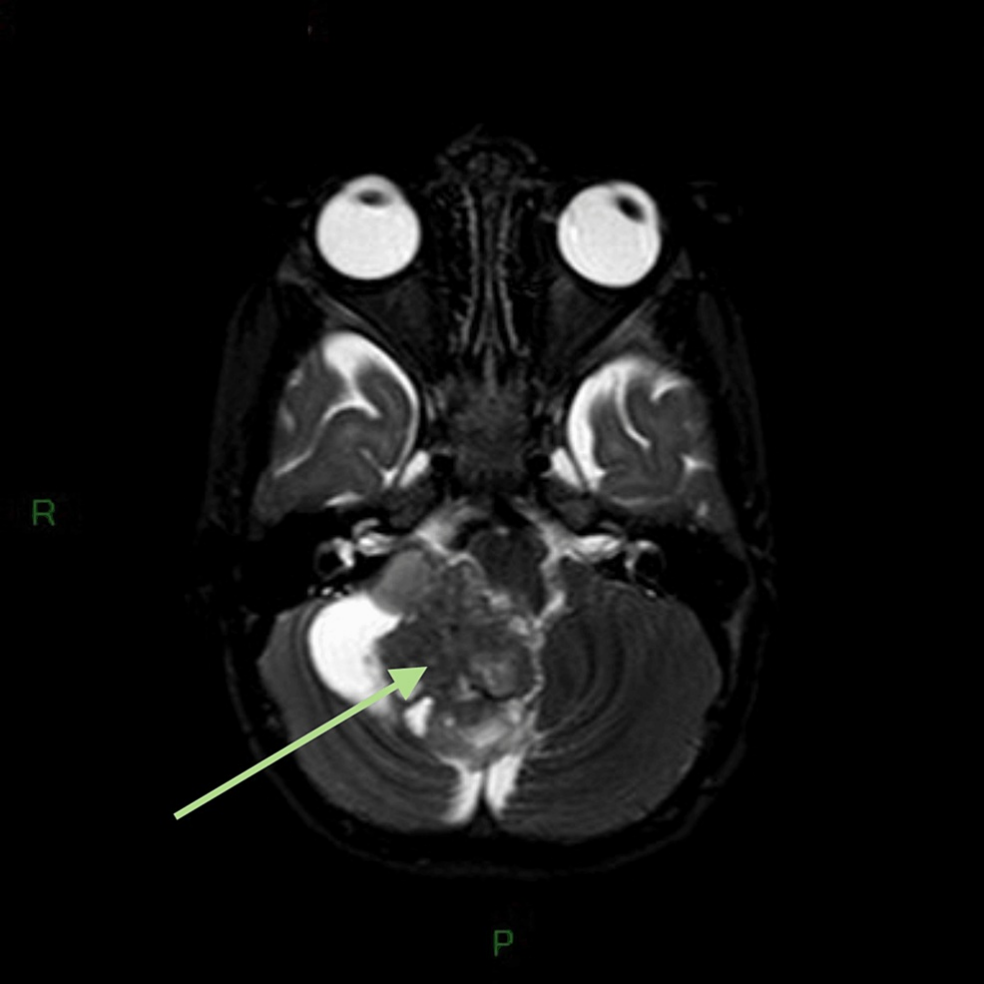
Preoperative axial T2-weighted STIR MRI. The image shows a round heterogeneous mass in the posterior fossa compressing the fourth ventricle with consequent obstructive hydrocephalus. R, right side; P, posterior; STIR, short-tau inversion recovery

At the age of 12 months, this young male patient was admitted to the hospital due to incoercive vomiting. An MRI revealed a 44-mm tumor of the posterior fossa with associated hydrocephalus (Figure 1). Complete surgical resection was performed, and histopathological analysis confirmed an AT/RT of the posterior fossa. Subsequently, the child underwent proton therapy in conjunction with adjuvant chemotherapy, including Vincristine, Cisplatin, Actinomycin D, and Cyclophosphamide. A total dose of 54 Gy was delivered in 30 fractions. The only early side effect of the radiation therapy was Grade 1 dermatitis. At the end of the treatment, the patient did not present any neurological deficits/impairments.

At the age of four, his parents suspected an auditory impairment, as their child exhibited sound localization difficulties. Consequently, the child was referred to our ENT department where a assessment of his hearing was carried out. Of note, former neonatal auditory screening with automated BAEP technique was normal. Additionally, before undergoing chemotherapy, the patient had a normal bilateral audiometric evaluation.

**Figure 2:**
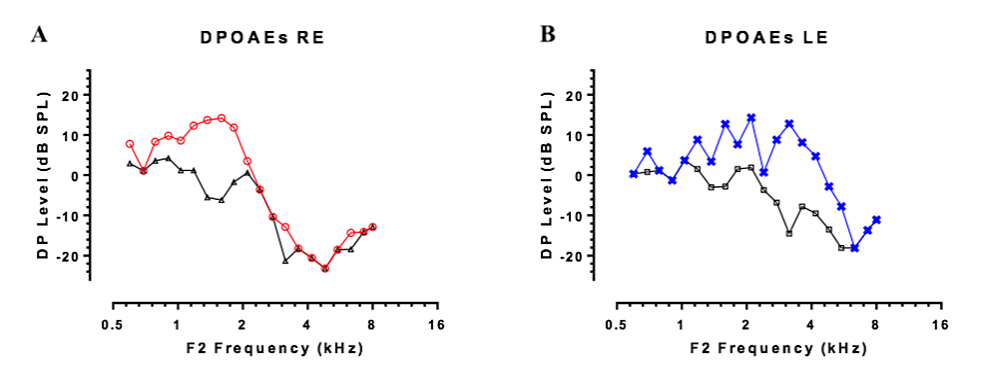
DPOAE results on the (A) right and (B) left sides. DPOAE, distortion-product otoacoustic emission; RE, right ear; LE, left ear

**Figure 3:**
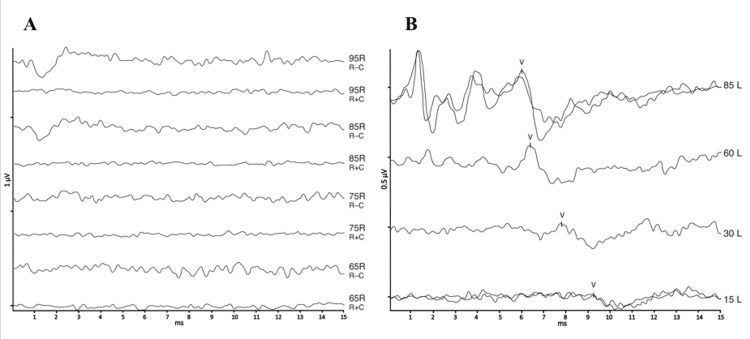
Brainstem auditory evoked potentials results on the (A) right and (B) left sides. On the brainstem auditory evoked potentials, none of the I-III-V waves were identified, but a cochlear microphonic potential was highlighted on the right side.

Pure-tone audiometry revealed a right-sided SNHL with a threshold of 75 dB HL, averaged across the frequencies of 500, 1,000, 2,000, and 4,000 Hz, with normal otomicroscopy and tympanometry. Evoked otoacoustic emissions and distortion-product otoacoustic emissions were present bilaterally (Figure 2). On the BAEP, none of the I-III-V waves were identified, but a cochlear microphonic potential was highlighted on the right side (Figure 3). 

The clinical neurological examination revealed limited cerebellar dysfunction, with mild right-hand dysmetria and difficulty in tandem gait. The clinical and instrumental vestibular evaluation (cervical sacculo-collic vestibular evoked myogenic potentials [cVEMPs] and a video-head impulse test [vHIT] of the six semicircular canals) was bilaterally normal. Posterior fossa MRI did not detect any vestibulo-cochlear nerve lesion. According to these results, a right PRT-induced ANSD diagnosis was suggested.

No auditory improvement was identified in the following audiological evaluations, with this unilateral hearing impairment remaining stable for the past eight years.

## Discussion

PBC is a common treatment for childhood cancer. Unfortunately, their use is burdened by short- and long-term side effects, such as hearing impairment. PBC-associated ototoxicity is related to the degeneration of mainly external hair cells as a consequence of oxidative stress and inflammation. HL is symmetric, bilateral, and sensorineural and affects first the higher frequencies (above 6,000 Hz). The deficit persists and, although it primarily manifests during treatment, HL can occasionally occur after chemotherapy discontinuation [2,7,8]. The risk of PBC-associated SNHL is further increased when oncological treatment requires its combination with XRT [9].

Interestingly, XRT, alone or in association with chemotherapy, has also been associated with SNHL, probably as a consequence of radiation-induced cochlear damage (if the cochlea is within the radiation field) [10]. These XRT-associated SNHL develop when the irradiation dose to the cochlea exceeds 40 Gy [11]. XRT-related ototoxicity is often unilateral since higher radiation dosage rarely implies both ears. The consecutive SNHL appears to be the result of outer hair cells and stria vascularis damage [1,12]. A previous study demonstrates that the BAEP latencies are not affected by XRT, suggesting that the hearing damage is located at the cochlear rather than retro-cochlear level [13].

PRT is increasingly used in pediatric radiation oncology. The unique radiation dose deposition reduces the volume of healthy tissues receiving medium-to-low radiation doses, thereby reducing the potential risk of long-term toxicities and second primary cancer development in comparison with XRT [14,15]. However, the late effects of PRT-related radiation are still largely unknown in the literature. Up to now, the only neuropathic side effects of PRT were optic neuropathies that typically develop several months or years after treatment discontinuation. Surprisingly, optic toxicities highlighted on visually evoked potentials increase with the distance of the target irradiated area. These results confirm uncertainties regarding the relative biological effectiveness of proton therapy, particularly due to the more heterogeneous linear energy transfer, especially in areas near the target volumes [16,17].

ANSD is rarely identified in the hearing impairment occurring with head and neck cancer treatment. Rare cases of ANSD have been described after vincristine administration, all of which were bilateral and reversible within a few months after the completion of treatment [18,19].

In this clinical case, the presence of evoked otoacoustic emissions and cochlear microphonic potential indicates that the function of the cochlea’s outer hair cells was preserved. However, no identifiable waves I, III, and V were recorded in the BAEP, suggesting the diagnosis of ANSD. Most cases of unilateral ANSD are congenital and are detected early through neonatal hearing screening. They are usually associated with cochlear nerve aplasia. In this case, a congenital origin can be ruled out, since the newborn hearing screening using automated BAEP testing was normal. Furthermore, the unilateral and nonreversible nature of the ANSD does not support the hypothesis of vincristine toxicity. Clinical and vestibular examinations have not revealed any involvement of other cranial nerves, including the vestibular nerves. We, therefore, suggest that this clinical case is the first description of a PRT-induced ANSD. Given that audiometric monitoring during oncological treatments often depends on pure-tone audiometry and OEAP, particularly in children, our observation suggests that, especially in the case of PRT of the cephalic segment, an additional BAEP evaluation should be considered, especially when there are symptoms and/or parental concerns. Detecting these deficits is important for enhancing patients' quality of life, especially in children [20].

## Conclusions

Auditory deficits are a common complication of cephalic segment oncological treatment. Pathophysiologically, ANSD rarely accounts for posttreatment hearing impairments in these cancer cases. The uniqueness of the case we describe lies in the causal association between proton therapy and the development of unilateral ANSD. To the best of our knowledge, this is the first reported instance of such a case in the medical literature.
